# Resting motor threshold in navigated transcranial magnetic stimulation: relationship between inter-individual variance and distinct clinical and anatomical factors

**DOI:** 10.1007/s00701-025-06726-x

**Published:** 2025-12-01

**Authors:** Felipe Monte Santo, Heike Schneider, Tizian Rosenstock, Ismael Moser, Maren Denker, Peter Vajkoczy, Thomas Picht, Melina Engelhardt

**Affiliations:** 1 Present Address: Department of Neurosurgery, Charité - Universitätsmedizin, Corporate Member of Freie Universität Berlin and Humboldt-Universität Zu Berlin, Charitéplatz 1, 10117 Berlin, Germany; 2 Present Address: Department of General Neurosurgery, Uniklinik Köln, Kerpener Str. 62, 50937 Cologne, Germany; 3 Berlin Institute of Health at Charité – Universitätsmedizin Berlin, BIH Biomedical Innovation Academy, BIH Charité Digital Clinician Scientist Program, Charitéplatz 1, 10117 Berlin, Germany; 4 Cluster of Excellence Matters of Activity. Image Space Material, Humboldt-Universität Zu Berlin, Unter Den Linden 6, 10099 Berlin, Germany; 5 Department of Neuroscience and Biomedical Engineering, Aalto University School of Science, Rakentajanaukio 2, 02150 Espoo, Finland

**Keywords:** Transcranial magnetic stimulation, Resting motor threshold, Preoperative mapping, Navigated brain stimulation

## Abstract

**Background:**

Navigated transcranial magnetic stimulation (nTMS) is increasingly used in neurosurgical practice for preoperative motor mapping. The resting motor threshold (RMT), a measure of cortical excitability, has been linked to postoperative motor outcomes. However, RMT is influenced by many inter-individual factors, potentially limiting its interpretability. This study aimed to assess the influence of clinical and anatomical variables on RMT variability in neurosurgical patients.

**Methods:**

A total of 642 patients with motor-eloquent brain lesions underwent preoperative nTMS, yielding 1,193 bilateral RMT observations. Variables included age, sex, tumor volume, peritumoral edema, tumor side, skull-to-cortex distance (SCD), recurrence, motor deficits, tumor dominance, handedness, histology, anatomical location, and use of anticonvulsants, benzodiazepines, corticosteroids, or antidepressants. Linear mixed models were applied.

**Results:**

RMT showed substantial inter-individual variability (mean 34 ± 8%, range 15–86%). Higher RMT included smaller peritumoral edema (estimate: -0.01; 95% CI: **-**0.03, -0.001; *p* = 0.032), greater SCD (estimate: 0.85; 95% CI: 0.63, 1.09; *p* < 0.001) and presence of motor deficits (estimate: 2.26; 95% CI: 0.89, 3.64; *p* = 0.001). Tumors outside the central region were associated with lower RMT (estimate: -1.87; 95% CI: -3.26, -0.47; *p* = 0.010). Medication analysis revealed that carbamazepine (estimate: 3.82; 95% CI: 0.81, 6.87; *p* = 0.014), benzodiazepines (estimate: 3.45; 95% CI: 1.11, 5.78; *p* = 0.004), and corticosteroids increased RMT (estimate: 1.56; 95% CI: 0.03, 3.09; *p* = 0.049), whereas antidepressants decreased it (estimate: -3.24; 95% CI: -5.90, -0.58; *p* = 0.019). Other factors showed no statistically significant effect.

**Conclusion:**

This study modeled the influence of clinical and anatomical factors on corticospinal excitability. This highlights the need for consideration of these variables when interpreting intervention-related changes in RMT or for risk stratification. Notably, the detailed analysis of common neurosurgical medications on RMT is unprecedented, emphasizing the importance of considering these factors.

## Introduction

Transcranial magnetic stimulation (TMS) has emerged as a valuable tool for preoperative planning and risk stratification in patients with brain tumors [40, 57, 71, 74, 75, 90]. By integrating individual brain imaging and electromyography, navigated transcranial magnetic stimulation (nTMS) enables a detailed assessment of the motor network before surgery, allowing for the delineation of functional motor areas and quantification of tumor-induced reorganization [25].

A crucial parameter in TMS is the resting motor threshold (RMT), which serves as a surrogate for cortical excitability. Accurate estimation of RMT is crucial not only for effective stimulation but also for ensuring patient safety and minimizing adverse effects of overstimulation [24, 82, 99]. RMT is defined as the lowest stimulation intensity required to elicit a motor response in a resting muscle in at least 5 out of 10 trials [76, 77]. Several methods exist for its estimation, with the Rossini-Rothwell method (R-R method) and maximum likelihood threshold-hunting being among the most widely used, both demonstrating strong reliability in the literature [2, 19, 77].

Despite its clinical relevance, RMT exhibits substantial variability both within and between individuals [89, 100]. Multiple factors have been proposed to contribute to this variability. For instance, RMT is generally thought to increase with age, partly due to the increased skull-to-cortex distance (SCD) resulting from age-related brain atrophy [39, 80]. In brain tumor patients, factors such as tumor location, volume, presence of motor deficits, and peritumoral edema have been linked to its variability [41, 89]. Pharmacological factors have also been implicated. Sodium channel–blocking antiepileptic drugs (AEDs), such as carbamazepine, have been consistently associated with elevated RMT values. [5, 30, 43, 51, 54, 63, 67, 68, 91, 94, 106] Furthermore, some antidepressants (e.g., citalopram and clomipramine) have been linked to increased RMT, whereas most studies have found no direct effect of benzodiazepines on RMT. [6, 12, 28, 32, 37, 56, 73, 79] However, the impact of some commonly used drugs, such as levetiracetam remains controversial, with conflicting findings reported in the literature. [17, 67, 68, 72, 87–89] Additionally, previous studies mostly analyzed the effect of these factors isolated, hence failing to account for patients taking multiple medications at the same time [5, 63, 106].

The present study aims to systematically evaluate the impact of multiple clinical and anatomical factors on RMT in a large cohort of over 600 brain tumor patients. We analyzed age, sex, presence of motor deficits, handedness, skull-to-cortex distance (SCD), and the intake of antiepileptic drugs (AEDs) as well as other medications with known or suspected neuromodulatory effects. In addition, we assessed tumor-related characteristics including location, side, hemispheric dominance, peritumoral edema, histology, recurrence, and tumor size. In contrast to earlier studies that examined smaller patient samples, we analyzed a substantially larger cohort, thereby providing a more comprehensive evaluation of factors influencing cortical excitability in the neurosurgical setting [17, 89]. With this broader dataset, we aimed to identify key variables associated with RMT variability, thereby contributing to the refinement of nTMS-based cortical mapping for clinical application.

## Methods

### Patients

**Fig. 1:**
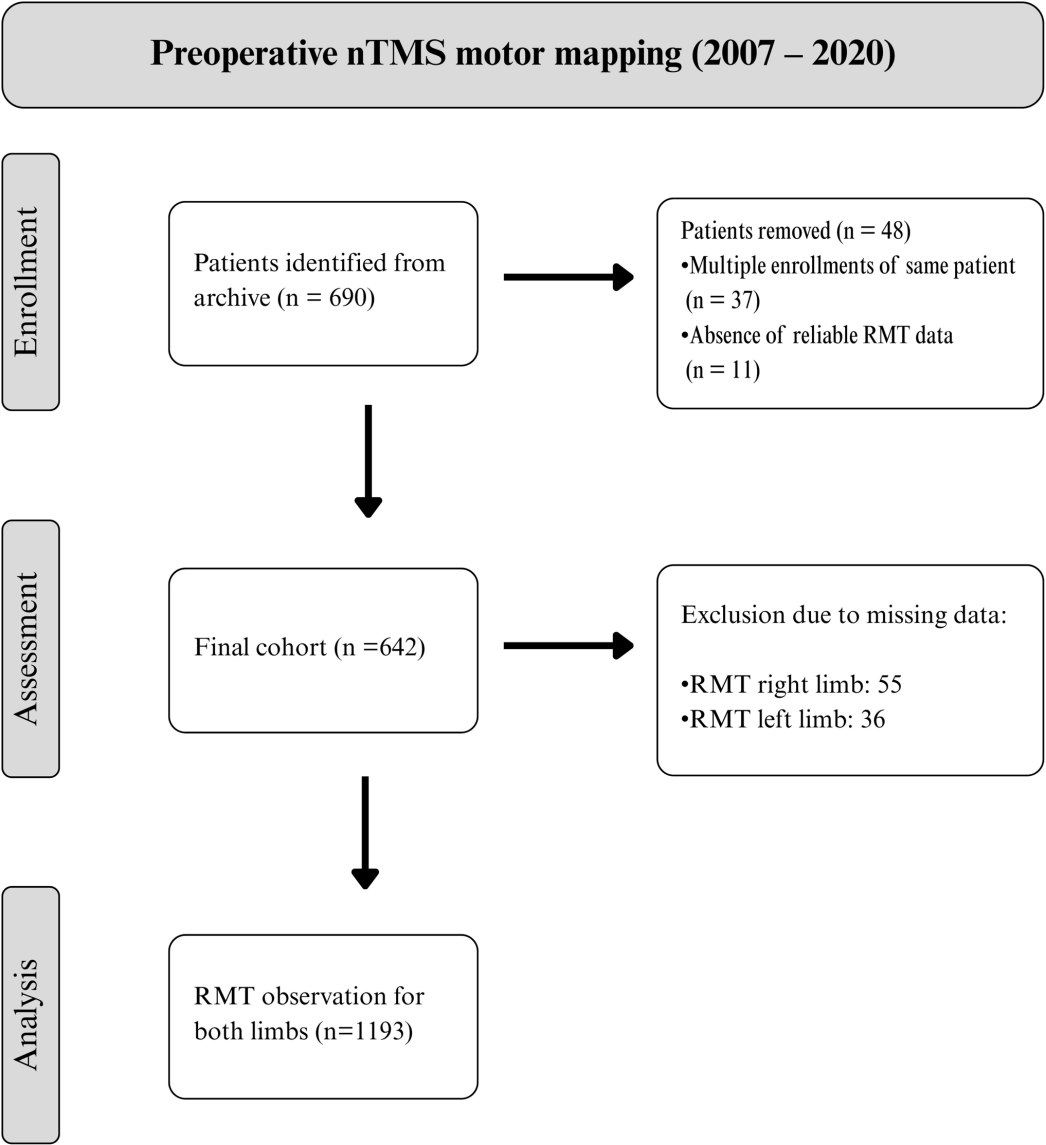
STROBE diagram illustrating patient selection and data processing

We conducted a retrospective analysis of patients who underwent preoperative nTMS assessment between 2007 and 2020. Inclusion criteria were: (I) age ≥ 18 years, (II) presence of a presumed motor-eloquent lesion on MRI, and (III) availability of a recorded RMT measurement. Our final cohort comprised 642 patients with at least one RMT measurement, resulting in 1193 RMT observations, as summarized in Fig. 1.

All patients provided written informed consent for preoperative nTMS. The retrospective analysis was approved by the local ethics committee of Charité – Universitätsmedizin Berlin (EA4/221/23) and the need for additional informed consent was waived. The study was conducted in accordance with the STROBE Guidelines and adhered to the principles of the Declaration of Helsinki and its later amendments. Study data were collected and managed using REDCap (Research Electronic Data Capture) [26, 27].

### Magnetic resonance imaging

As part of the clinical routine, all patients underwent structural brain MRI using either a 1.5 T or 3 T MRI scanner (GE Healthcare) with an 8-channel head coil. These MRI datasets were utilized for presurgical evaluation and served as an individual navigational reference for preoperative nTMS assessments.

For analysis, MRI review was performed using Brainlab Elements (Brainlab AG, Munich, Germany). Tumor recurrence was defined as radiological evidence of progression after surgical therapy. Tumor side was classified as left, right, or bilateral. Tumor location was defined as central (involving the precentral and/or postcentral gyri) or non-central (all other regions) to specifically investigate the role of Rolandic involvement. Finer anatomical classifications were avoided due to the high number of spatially extensive tumors spanning multiple areas.

Tumor and peritumoral edema volumes were manually measured on MRI and recorded in milliliters. Cases with unclear tumor and/or edema margins were excluded from volumetric analysis. For patients with multiple lesions, only tumors planned for resection were analyzed. SCD was measured in the Nexstim software (Nexstim, Helsinki, Finland) and recorded in millimeters.

### Preoperative nTMS assessment

During the nTMS examination, patients were seated comfortably and instructed to keep their hands relaxed and their eyes open. Motor mapping was conducted using a Nexstim eXimia or NBS5 system (Nexstim, Helsinki, Finland) with a figure-eight coil. Electromyographic responses of the first dorsal interosseous muscle were recorded via adhesive electrodes (Neuroline 720; Ambu, Copenhagen, Denmark) to determine the motor hotspot—defined as the site where optimal coil angulation and rotation consistently elicited the highest motor-evoked potentials (MEP).

RMT was determined using either the Rossini-Rothwell method or automated threshold hunting, depending on the year of assessment and examiner preference. RMT values were recorded as a percentage of the maximum stimulator output.

### Clinical and anatomical factors

Clinical data were extracted from electronic medical records. Medications were classified as AEDs, benzodiazepines, corticosteroids, antidepressants and other drugs. Within each pharmacological class, the differential effect of the most common drugs were also analyzed individually.

Handedness was assessed using the Edinburgh Handedness Inventory, and tumor dominance was inferred accordingly [60]. Motor weakness was defined as a score below 5 on the British Medical Research Council (MRC) scale for upper extremities [9].

Histopathological classification followed the 4th Edition (2007) or Revised 4th Edition (2016) of the WHO Classification of Tumors of the Central Nervous System, depending on the year of diagnosis [47, 48]. For statistical analysis, histological subtypes were grouped into five categories: Glioblastoma (GBM), Astrocytoma Grade 3, Other gliomas, Metastases and Other tumors.

### Statistical analysis

Normal distribution of each outcome was determined based on visual inspection. Accordingly, either mean and standard deviation or median and range are reported. Tables 1 and 2 show the sample characteristics and demographic statistics for clinical factors and medications respectively. The impact of each individual factor on the RMT was analyzed using separate linear mixed models with a random intercept for patients.

**Table 1:** Overview and independent analysis of the clinical and anatomical variables

Variable	Values	NA	Estimate/CI	P-value
RMT (Observations)	1193	91	-	-
Right limb	606	55	-	-
Left limb	587	36	-	-
RMT (Intensity in %)	34 ± 8	-	-	-
Age (years)	53.3 ± 15.6	-	0.01 (−0.02, 0.05)	0.412
**Edema (ml)**	**44.9 (0.3—210)**	**165**	**−0.02 (−0.03, −0.006)**	**0.005**
Tumor volume (ml)	14.1 (0.2—137)	5	−0.02 (−0.04, 0.003)	0.087
**SCD (mm)**	**14.8 ± 2.4**	**24**	**0.87 (0.66, 1.08)**	** < 0.001**
Gender	-	-	-	-
Female	285 (44.4%)	-	-	
Male	357 (55.5%)	-	0.13 (−1.3, 1.1)	0.826
Tumor side	-	-	-	-
Right	341 (53.1%)	-	-	-
Left	296 (46.1%)	-	0.47 (−0.75, 1.71)	0.449
Bilateral	5 (0.8%)	-	x	x
Handedness	-	300 (46.8%)	-	-
Right	322 (50.1%)	-	-	-
Left	15 (2.3%)	-	−0.99 (−4.81, 2.81)	0.608
Ambidextrous	5 (0.8%)	-	x	x
Dominant Tumor	-	300 (46.8%)	-	-
No	201 (31.3%)	-	-	-
Yes	141 (21.9%)	-	1.09 (−0.47, 2.65)	0.174
Histology	-	-	-	-
Astrocytoma 3	61 (9.5%)	-	-	-
GBM	217 (33.8%)	-	−1.93 (−4.15, 0.28)	0.088
Other Glioma	83 (12.9%)	-	0.16 (−2.40, 2.73)	0.901
Metastasis	161 (25.1%)	-	−0.71 (−3.01, 1.58)	0.543
Other	120 (18.7%)	-	0.12 (−2.29, 2.54)	0.920
Recurrence	-	6 (0.9%)	-	-
No	522 (81.3%)	-	-	-
Yes	114 (17.8%)	-	−1.35 (−2.96, 0.258)	0.1
**Deficit**		**2 (0.3%)**	**-**	**-**
** No**	**421 (65.6%)**	**-**	**-**	**-**
** Yes**	**219 (34.1%)**	**-**	**1.64 (0.35, 2.94)**	**0.013**
**Location**		**-**	**-**	**-**
** Central**	**358 (55.7%)**	**-**	**-**	**-**
** Non central**	**284 (44.3%)**	**-**	**−2.91 (−4.12, −1.69)**	** < 0.001**

Overview of clinical and anatomical variables. Estimates, 95% confidence intervals (CI) and p-values correspond to linear mixed model analyses evaluating the relationship between RMT and each variable independently. Values are presented as mean ± standard deviation for normally distributed variables, median (range) for non-normally distributed variables, and count (percentage) for categorical variables. Fields marked with “X” were excluded from statistical analysis due to small group size. Abbreviations: *RMT* Resting Motor Threshold, *SCD* Skull-to-Cortex Distance, *GBM* Glioblastoma; Astrocytoma 3 = Astrocytoma WHO Grade 3

**Table 2:** Overview and independent analysis of medication intake

Variable	Values	NA	Estimate/CI	P-value
Medication	494 (70.0%)	20 (3.1%)	0.77 (−0.77, 2.32)	0.330
AED	296 (46.1%)	21 (3.3%)	0.91 (−0.35, 2.171)	0.158
AED medication*	-	-	-	-
Levetiracetam	233	-	-	-
Carbamazepine	26	-	-	-
Lamotrigine	22	-	-	-
Lacosamide	20	-	-	-
Other	57	-	-	-
Benzodiazepine*	**58 (9%)**	**29 (4.5%)**	**5.04 (2.91, 7.17)**	** < 0.001**
Benzodiazepine medication	-	-	-	-
Clobazam	27	-	-	-
Lorazepam	17	-	-	-
Other	16	-	-	-
Corticoid	314 (49%)	39 (6%)	−0.19 (−1.45, 1.07)	0.765
Corticoid medication	-	-	-	-
Dexamethasone	304	-	-	-
Other	10	-	-	-
Antidepressant	44 (6.8%)	29 (4.5%)	−1.15 (−3.64, 1.34)	0.366
Antidepressant medication*	-	-	-	-
Citalopram	14	-	-	-
Other	39	-	-	-
Other medication	30 (4.7%)	29 (4.5%)	1.70 (−1.23, 4.63)	0.256
Other medication (specified)	-	-	-	-
Zolpidem	13	-	-	-
Zopiclone	9	-	-	-

Overview of medication intake. Estimates, 95% confidence intervals (CI) and p-values correspond to linear mixed model analyses evaluating the relationship between RMT and each medication class independently. The reference category for each variable is “no intake” of the respective medication. Values are presented as number of patients and percentage. Entries marked with an asterisk (*) indicate deviations in patient total counts due to intake of multiple medications by individual patients. Abbreviations: *RMT* Resting Motor Threshold, *AED* Antiepileptic Drug

**Fig. 2:**
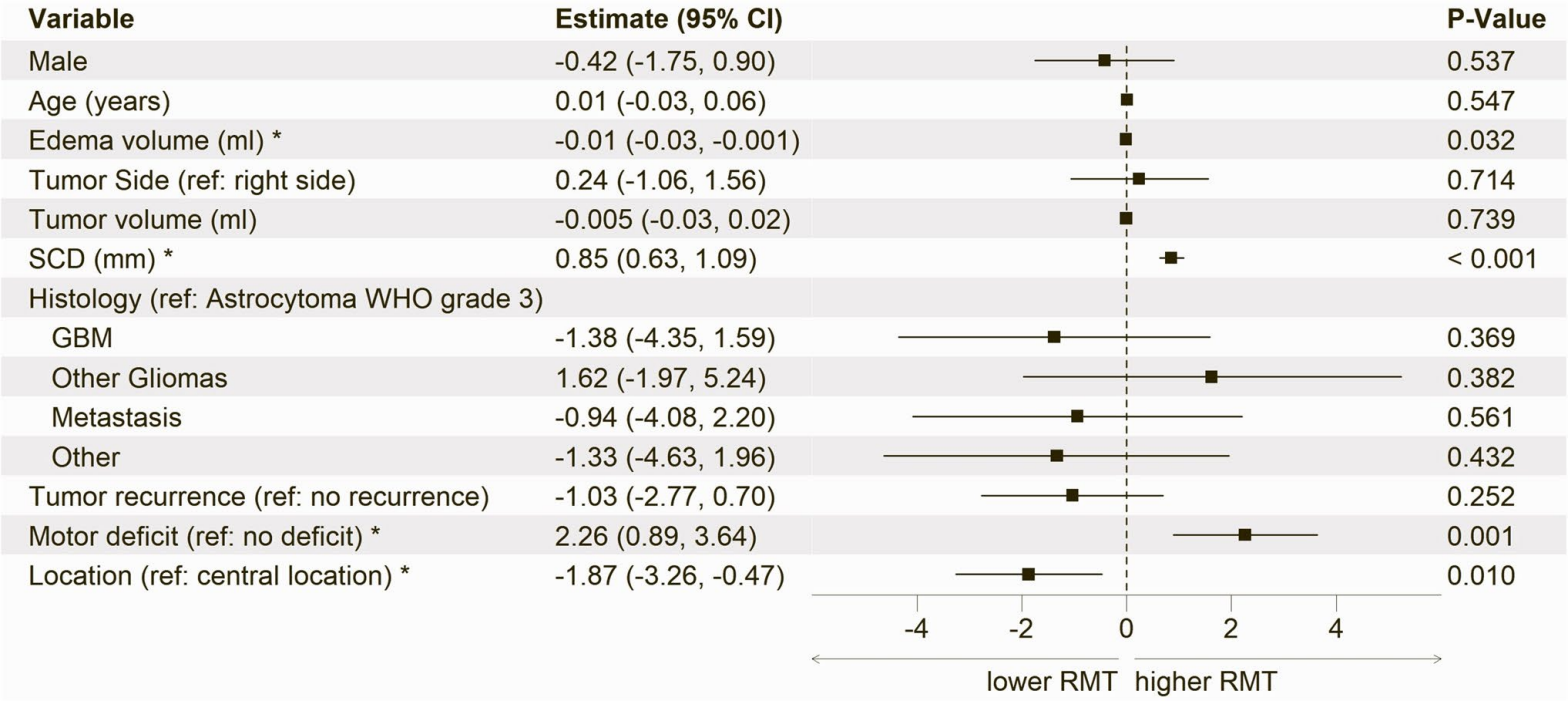
Base model showing estimates, 95% confidence intervals (CI) and p-values from linear mixed model analyses of the association between RMT and clinical or anatomical variables. The model explained 13.1% of the variance in RMT (R^2^ = 0.131). * Statistically significant (*p* < 0.05). Abbreviations: RMT = Resting Motor Threshold; SCD = Skull-to-Cortex Distance; GBM = Glioblastoma

Subsequently, we developed a combined linear mixed model (base model) with a random intercept for patients and incorporating all variables except medications (Fig. 2). This model was designed to benchmark our results against the models introduced by Sollmann et al. and Eibl et al. in a large cohort.

**Fig. 3:**
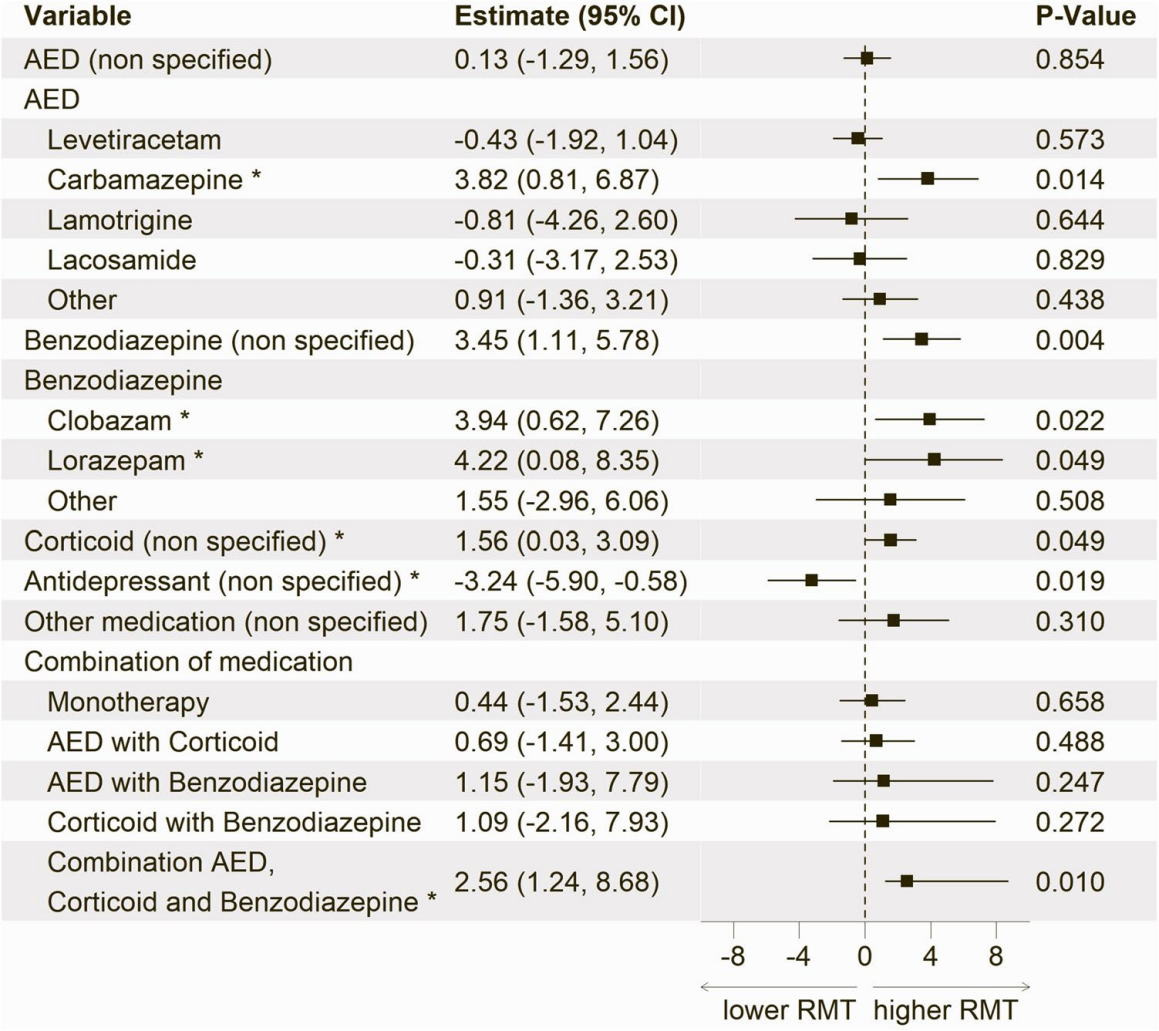
Medication models showing estimates, 95% confidence intervals (CI) and p-values from linear mixed model analyses of the association between RMT and medication intake. Each drug or drug class was added separately to the base model described in Fig. 2. The different models are divided by a bold line. The reference category for each variable is “no intake” of the respective medication. * Statistically significant (p < 0.05). Abbreviations: RMT = Resting Motor Threshold; AED = Antiepileptic Drug

To evaluate the impact of individual medications on the RMT, each class of medications was added separately to the previous base model. For AEDs and benzodiazepines, additional models were run including specific medication types. Finally, a model assessed the effect of the most frequent combination of medications to account for the common intake of multiple different drugs (Fig. 3). All medication models were designed exploratory to identify potential pharmacological influences on excitability in a real-world, heterogeneous neurosurgical population. Accordingly, we did not apply formal correction for multiple comparisons, consistent with the hypothesis-generating nature of these analyses.

Normality of residuals was assured by visual inspection of Q-Q Plots for all models. Variance inflation factor analysis confirmed the absence of relevant multicollinearity among variables in all models.

We used a two-sided significance level of α = 0.05 and no adjustment for multiple testing was applied in this analysis.

All statistical analyses were conducted using R version 4.2.2 (*The R Foundation for Statistical Computing, Vienna, Austria*). The following R packages were utilized: *readr, tidyr, dplyr, forcats, ggplot2, lmerTest, car, r2glmm, grid, forestplot* and *forestploter*. [21, 23, 35, 42, 70, 81, 102]

## Results

Our study included 642 patients with a total of 1,193 RMT observations from both hemispheres and exhibited substantial inter-individual variability (mean: 34 ± 8%, range: 15–86). Patients had a mean age of 53.3 ± 15.6 years (range: 19–85), and a median tumor volume of 14.1 ml (range: 0.2–137), while the mean SCD measured 14.8 ± 2.4 mm (range: 8.5–28.1). Among the participants, 285 (44.4%) were female. Tumors were located on the right side in 341 cases (53.1%), on the left in 296 cases (46.1%), and bilaterally in 5 cases (0.8%). The most frequent histological types included GBM (33.8%), metastases (25.1%), and other gliomas (12.9%). Motor deficits were observed in 34.1% of patients. Regarding medication, 70% of patients were receiving some form of pharmacological treatment, with 46.1% receiving AEDs. Benzodiazepines were used by 9% of patients, while 6.8% took antidepressants. Corticosteroids were administered in 49% of cases. These results are summarized in Table 1 and Table 2.

### Effect of anatomical and clinical variables on RMT

The base model (Fig. 2) revealed a significant inverse relationship between peritumoral edema volume and RMT, where greater edema volumes were associated with lower RMT values (estimate: −0.01; 95% CI: **-**0.03, −0.001; *p* = 0.032). In contrast, SCD showed a direct relationship with RMT (estimate: 0.85; 95% CI: 0.63, 1.09; *p* < 0.001). Additionally, the presence of motor deficits (estimate: 2.26; 95% CI: 0.89, 3.64; *p* = 0.001) was associated with higher RMT values, whereas tumors located outside the central region were associated with lower RMT values (estimate: −1.87; 95% CI: −3.26, −0.47; *p* = 0.010). Conversely, sex, age, tumor volume, tumor side, recurrence, tumor dominance, handedness, and histology had no statistically significant effect on RMT. Independent analyses of these variables yielded no substantial differences compared to the base model (Table 1).

### Effect of medications on RMT

The exploratory analysis of medication effects on RMT (Fig. 3) revealed significant associations with specific drug classes and their combinations. While the general use of AEDs did not significantly alter RMT values, the examination of individual medications showed that carbamazepine intake was associated with a notable increase in RMT (3.82 [0.81, 6.87], *p* = 0.014). Other AEDs, including levetiracetam, lamotrigine, and lacosamide, did not demonstrate a significant effect. Benzodiazepines were also linked to increased RMT values (3.45 [1.11, 5.78], *p* = 0.004), with clobazam (3.94 [0.62, 7.26], *p* = 0.022) and lorazepam (4.22 [0.08, 8.35], *p* = 0.049) showing significant individual associations, although weaker than the overall benzodiazepine effect.

Corticosteroid use exhibited an association with higher RMT (1.56 [0.03, 3.09], *p* = 0.049). In contrast, antidepressants were the only medication class associated with a decrease in RMT (−3.24 [−5.90, −0.58], *p* = 0.019). Notably, although antidepressants and corticosteroids exhibited a significant effect in the mixed medication model, this effect was not reproduced when they were analyzed independently (Table 2).

When evaluating medication combinations, the concurrent use of AEDs, corticosteroids, and benzodiazepines resulted in a significant increase in RMT (2.56 [1.24, 8.68], *p* = 0.010), whereas other combinations, such as AEDs with corticosteroids or benzodiazepines, did not show a significant effect.

## Discussion

In this study, we investigated the influence of various clinical and anatomical factors on RMT variability in neurosurgical patients undergoing nTMS.

### Impact of clinical factors

Our study observed a strong direct relationship between SCD and RMT, reinforcing previous findings in the literature [29, 39, 52, 93]. This effect is likely attributable to the physical properties of electromagnetic stimulation, where field strength decreases with distance, as explained by Coulomb’s inverse-square law [98]. Age, however, did not significantly affect RMT in our study. Previous studies have linked age to changes in cortical excitability, possibly mediated by increased skull to cortex distance due to brain atrophy or by degeneration of spinal motor neurons, but overall evidence remains inconsistent, with some reports finding an inverse correlation and others no effect [8, 17, 61, 64, 65, 78, 84, 89, 103].

In contrast to the findings of Sollmann et al., who reported a potential association between female sex and higher RMT, we did not observe a significant relationship between RMT and sex in our cohort [89]. However, a significant sex-related effect on RMT has not been consistently demonstrated. [34, 65, 80, 85, 86] This discrepancy may reflect differences in hormonal status, which were not studied in the present study, since estradiol has been associated with excitatory effects, while progesterone appears to exert inhibitory effects.

Motor deficits were strongly associated with increased RMT, consistent with previous findings that impaired corticospinal function correlates with higher motor thresholds [17, 89]. This likely reflects reduced cortical excitability due to tumor-related factors, such as infiltration or compression of the corticospinal tract. Similarly, studies in patients with ischemic motor deficits have demonstrated higher RMTs, suggesting that disrupted motor pathways and compensatory cortical reorganization contribute to this effect [3, 96, 97].

In line with previous literature, we observed an inverse correlation between RMT and peritumoral edema volume, supporting the hypothesis that edema-induced fiber orientation changes and neuronal osmolality alterations may underlie this effect. [17, 29, 44, 59, 89]

In contrast, tumor volume did not significantly impact RMT in our study, aligning with findings from Sollmann et al. [89] However, Eibl et al. reported higher RMT values with larger lesions [17]. Given that tumor volume alone does not necessarily reflect spatial proximity to motor-eloquent areas, we consider our results to be expected. Conversely, this was likely not the case for edema, as peritumoral edema extends beyond the tumor margins and may involve motor-eloquent regions even when the tumor itself does not, thereby exerting a more widespread anatomical effect on RMT.

Our study demonstrated higher RMTs in central lesions compared to non-central tumors, consistent with the hypothesis that direct infiltration of motor-eloquent areas alters cortical excitability. The influence of tumor location on RMT has been inconsistently reported. Eibl et al. observed higher RMTs in lesions infiltrating the precentral gyrus but lower RMTs when involving the postcentral gyrus [17]. Conversely, Sollmann et al. found lower RMTs in central-region tumors [89]. These partially conflicting findings could be explained by individual tumor characteristics or methodological differences in the anatomical classification of the lesions.

While previous studies suggested a relationship between tumor histology, WHO grade, and RMT, the underlying pathophysiological mechanisms remain unclear [17, 45, 89]. In contrast, our findings did not demonstrate a significant association between histological subtype and RMT values. Although detailed molecular stratification was not performed in the present study, a recent investigation from our center analyzing molecular and histopathological tumor profiles in a similar cohort, likewise reported no correlation between absolute RMT and tumor histology, further supporting these results. However, it found that gliomas, compared to benign lesions, and IDH–wildtype glioblastomas, compared to IDH–mutant gliomas, more frequently exhibited pathologic interhemispheric RMT ratios (i.e., affected/healthy hemispheres), indicating a greater degree of excitability imbalance in more aggressive tumor subtypes [58].

### Influence of medications

The pharmacological modulation of RMT is well-documented, with numerous studies demonstrating drug-induced changes in cortical excitability. [5, 63, 106] The potential influence of medications on RMT has led to recommendations for re-evaluating RMT after medication changes, based on the theoretical risk of TMS-induced seizures, despite a lack of clear evidence supporting this relationship [46, 69]. Although most previous studies have precisely examined the effects of individual drugs on RMT, the concurrent use of multiple medications in neurosurgical patients presents a unique challenge [5, 63, 106].

### *Antiepileptic drugs*

AEDs exhibit diverse pharmacological mechanisms, and as a group, AEDs did not significantly affect RMT. Due to incomplete documentation in older records, we did not differentiate between prophylactic and therapeutic AED use nor clinical response to therapy (i.e. seizure control).

Our study found no significant relationship between RMT and levetiracetam. This drug is unique among AEDs as it modulates synaptic vesicle protein 2 A [49, 66, 95]. Studies have reported conflicting effects of levetiracetam on RMT, with findings ranging from increased, decreased, or unchanged RMT [17, 67, 68, 72, 87–89]. Differences in the methodology of studies could be the reason for such a wide discrepancy across the results in literature. Duration of treatment, for example, most likely plays a relevant role [38, 51, 89].

Voltage-gated sodium channel (VGSC) blockers, such as carbamazepine, lamotrigine, and lacosamide, have been shown to increase RMT in prior studies. [6, 30, 43, 54, 63, 67, 68, 91, 94, 105, 106] Our results were consistent with this relationship for carbamazepine but did not show a significant effect of lamotrigine or lacosamide on RMT. Our small sample of patients taking these drugs could be a possible cause for this discrepancy with the literature.

The observed differences in RMT modulation between levetiracetam and VGSC blockers likely stem from their distinct mechanisms of action. VGSC blockers exert their effects by stabilizing voltage-gated sodium channels in their inactive state, directly limiting neuronal excitability and raising the threshold required to generate an action potential [1, 50]. This direct inhibition of neuronal firing reliably increases RMT across multiple studies [6, 30, 43, 54, 63, 67, 68, 91, 94, 105, 106]. In contrast, levetiracetam does not directly alter membrane excitability but instead modulates synaptic transmission by reducing presynaptic glutamate release through SV2A interaction and calcium channel inhibition [10, 11, 107]. While this mechanism decreases excitatory drive, its impact on RMT is more variable since RMT primarily reflects the intrinsic excitability of motor cortex neurons rather than synaptic activity [18, 83]. This distinction could explain why sodium channel blockers elevate RMT, whereas levetiracetam's effect remains inconsistent across studies.

### *Benzodiazepines*

Our findings showed increased RMT in patients taking benzodiazepines, particularly clobazam and lorazepam. Benzodiazepines act as γ-aminobutyric acid type A (GABAA) receptor agonists, with well-documented reductions in MEP amplitudes after the intake of midazolam, lorazepam or diazepam [6, 12, 28, 32, 37, 79]. While most studies indicate that benzodiazepines do not affect RMT after a single intake in the usual therapeutic dose [12, 14, 33, 37, 91, 104], at least one study has demonstrated an increase in RMT following chronic diazepam use, as well as two cases of overdoses [62]. Given that, in neurosurgical patients, benzodiazepines are frequently administered not as isolated single doses but rather repeatedly (e.g., delirium, prolonged agitation), our results align with the previous report concerning chronic use, albeit the limited number of studies in this population [7, 36, 62].

### *Corticosteroids*

Our study found a weak association between corticosteroid intake and higher RMT values, whereas previous studies have suggested that corticosteroids may enhance cortical excitability [4, 55]. Furthermore, methylprednisolone has been shown to reduce RMT in multiple sclerosis patients, though this effect may be related to the underlying disease rather than the drug itself [20]. Considering this drug is used in brain tumor patients almost exclusively to treat brain edema, we believe our findings to be consistent with the known effects of corticosteroids in reducing brain edema, which itself decreases RMT [15].

### *Antidepressants*

Lower RMT values were observed during antidepressant intake in our study, while previous studies have shown mixed results. Some selective serotonin reuptake inhibitors (SSRI), such as fluoxetine and sertraline, appear to have no effect on RMT, whereas citalopram (also a SSRI) and clomipramine (tricyclic antidepressant) have been associated with increased RMT. [22, 31, 53, 56, 73, 101] This discrepancy may stem from the varied mechanisms of action among different antidepressant subclasses, which could not be accounted for in our study due to the small number of patients taking antidepressants.

### *Other medications*

The "other drugs" category, composed primarily of non-benzodiazepine hypnotics ("Z-drugs," such as zolpidem and zopiclone), did not significantly influence RMT. These drugs act on GABAA receptors, similar to benzodiazepines, but differ structurally and pharmacologically [16]. Acute GABAA receptor agonism is not expected to alter RMT, and the literature also demonstrated that zolpidem does not affect RMT [13]. Although it is common that many patients receive hypnotic drugs during hospitalization, our study did not have detailed treatment duration data for these medications [92].

#### Clinical implications

In summary, these findings emphasize that RMT values should not be interpreted in isolation, but rather in the context of clinical and anatomical factors that can modulate cortical excitability. Integrating this knowledge may improve the precision of RMT assessment and, in turn, enhance the reliability of nTMS-based motor mapping for preoperative risk stratification and surgical planning.

Prior works have demonstrated that preoperative RMT values, particularly interhemispheric RMT ratios or elevated absolute thresholds, can predict postoperative motor outcomes in glioma surgery. The present study offers complementary insights by identifying possible factors that systematically influence RMT at the time of mapping [57, 74, 75]. These findings warrant consideration when incorporating RMT into prognostic models, to ensure valid interpretation and clinical applicability.

#### Limitations

This study's retrospective design introduces inherent limitations, including potential biases from incomplete documentation of medication use and clinical histories. Although medication intake was examined, details such as treatment duration, treatment dose and cumulative effects were not fully accounted for, and interactions between medications could not be isolated. Moreover, some medication subgroups were also relatively small (e.g., benzodiazepines 9%, antidepressants 6.8%), limiting statistical power. These low frequencies, however, also reflect the clinical reality of neurosurgical populations, in which benzodiazepines and antidepressants are prescribed far less often than antiepileptic drugs or corticosteroids. Further, the between-subject design of our study allowed for the analyses of a large cohort of patients with varying medications, however, does not allow for inference of direct causal effects of the drugs as a within-subject design would have. Additionally, variations in nTMS protocols over the 13-year study period, including inter-examiner differences and treatment protocols, may have introduced methodological inconsistencies. Finally, no correction for multiple comparisons was applied to the exploratory medication models. These analyses were hypothesis-generating by design and should be interpreted with appropriate caution until validated in future confirmatory studies.

## Conclusions

In the present study, we demonstrated that the RMT exhibits substantial inter-individual variability among neurosurgical patients. Our analysis identified several statistically significant associations with higher RMT values: smaller volume of peritumoral edema, greater skull-to-cortex distance, presence of motor deficits, and central tumor location. Furthermore, medication analysis revealed that carbamazepine, benzodiazepines, and corticosteroids were associated with increased RMT, while antidepressants were associated with decreased RMT.​

Due to the hypothesis-generating nature of our findings, further independent replication is required. Future prospective work should test these associations with prespecified adjustment for multiplicity. Nonetheless, this study is strengthened by its large sample size, allowing for a comprehensive evaluation of RMT variability in neurosurgical patients, adjusted for possible confounding variables. Notably, the detailed analysis of common neurosurgical medications on RMT is unprecedented, highlighting the importance of considering these factors. Given the high inter-individual variability of RMT, consideration of these factors is crucial for accurate, precise, and safe nTMS examinations. While these insights are particularly relevant for cortical mapping in preoperative planning, they may also be applicable to other nTMS modalities.

## Data Availability

No datasets were generated or analysed during the current study.

## Abbreviations

AED: Antiepileptic Drug
GABAA: γ-Aminobutyric acid type A
GBM: Glioblastoma
MEP: Motor Evoked Potential
MRC scale: British Medical Research Council´s scale
MRI: Magnetic Resonance Imaging
nTMS: Navigated Transcranial Magnetic Stimulation
RMT: Resting Motor Threshold
SCD: Skull-to-Cortex Distance
SSRI: Selective Serotonin Reuptake Inhibitor
TMS: Transcranial Magnetic Stimulation
VGSC: Voltage-gated sodium channel
WHO: World Health Organization

## References

[1] Abdelsayed M, Sokolov S (2013) Voltage-gated sodium channels: pharmaceutical targets via anticonvulsants to treat epileptic syndromes. Channels (Austin) 7:146–152. 10.4161/chan.24380 23531742 10.4161/chan.24380 PMC3710341

[2] Awiszus F (2003) TMS and threshold hunting. Suppl Clin Neurophysiol 56:13–23. 10.1016/s1567-424x(09)70205-3 14677378 10.1016/s1567-424x(09)70205-3

[3] Bastings EP, Greenberg JP, Good DC (2002) Hand motor recovery after stroke: a transcranial magnetic stimulation mapping study of motor output areas and their relation to functional status. Neurorehabil Neural Repair 16:275–282. 10.1177/154596802401105207 12234089 10.1177/154596802401105207

[4] Baudry S, Lanfranco F, Merletti R, Duchateau J, Minetto MA (2014) Effects of short-term dexamethasone administration on corticospinal excitability. Med Sci Sports Exerc 46:695–701. 10.1249/MSS.0000000000000162 24051659 10.1249/MSS.0000000000000162

[5] Boroojerdi B (2002) Pharmacologic influences on TMS effects. J Clin Neurophysiol 19:255–271. 10.1097/00004691-200208000-00002 12436084 10.1097/00004691-200208000-00002

[6] Boroojerdi B, Battaglia F, Muellbacher W, Cohen LG (2001) Mechanisms influencing stimulus-response properties of the human corticospinal system. Clin Neurophysiol 112:931–937. 10.1016/s1388-2457(01)00523-5 11336911 10.1016/s1388-2457(01)00523-5

[7] Chen L, Xu M, Li GY, Cai WX, Zhou JX (2014) Incidence, risk factors and consequences of emergence agitation in adult patients after elective craniotomy for brain tumor: a prospective cohort study. PLoS ONE 9:e114239. 10.1371/journal.pone.0114239 25493435 10.1371/journal.pone.0114239 PMC4262354

[8] Clark BC, Taylor JL (2011) Age-related changes in motor cortical properties and voluntary activation of skeletal muscle. Curr Aging Sci 4:192–199. 10.2174/1874609811104030192 21529329 10.2174/1874609811104030192 PMC3184350

[9] Compston A (2010) Aids to the investigation of peripheral nerve injuries. Medical research council: nerve injuries research committee. His Majesty’s Stationery Office: 1942; pp. 48 (iii) and 74 figures and 7 diagrams; with aids to the examination of the peripheral nervous system. By Michael O’Brien for the Guarantors of Brain. Saunders Elsevier: 2010; pp. [8] 64 and 94 figures. Brain 133:2838–2844. 10.1093/brain/awq270 20928945 10.1093/brain/awq270

[10] Contreras-Garcia IJ, Cardenas-Rodriguez N, Romo-Mancillas A, Bandala C, Zamudio SR, Gomez-Manzo S, Hernandez-Ochoa B, Mendoza-Torreblanca JG, Pichardo-Macias LA (2022) Levetiracetam mechanisms of action: from molecules to systems. Pharmaceuticals (Basel). 10.3390/ph15040475 35455472 10.3390/ph15040475 PMC9030752

[11] Contreras-Garcia IJ, Gomez-Lira G, Phillips-Farfan BV, Pichardo-Macias LA, Garcia-Cruz ME, Chavez-Pacheco JL, Mendoza-Torreblanca JG (2021) Synaptic vesicle protein 2A expression in glutamatergic terminals is associated with the response to levetiracetam treatment. Brain Sci. 10.3390/brainsci11050531 33922424 10.3390/brainsci11050531 PMC8145097

[12] Di Lazzaro V, Oliviero A, Meglio M, Cioni B, Tamburrini G, Tonali P, Rothwell JC (2000) Direct demonstration of the effect of lorazepam on the excitability of the human motor cortex. Clin Neurophysiol 111:794–799. 10.1016/s1388-2457(99)00314-4 10802448 10.1016/s1388-2457(99)00314-4

[13] Di Lazzaro V, Pilato F, Dileone M, Profice P, Ranieri F, Ricci V, Bria P, Tonali PA, Ziemann U (2007) Segregating two inhibitory circuits in human motor cortex at the level of GABAA receptor subtypes: a TMS study. Clin Neurophysiol 118:2207–2214. 10.1016/j.clinph.2007.07.005 17709293 10.1016/j.clinph.2007.07.005

[14] Di Lazzaro V, Pilato F, Dileone M, Tonali PA, Ziemann U (2005) Dissociated effects of diazepam and lorazepam on short-latency afferent inhibition. J Physiol 569:315–323. 10.1113/jphysiol.2005.092155 16141274 10.1113/jphysiol.2005.092155 PMC1464195

[15] Dietrich J, Rao K, Pastorino S, Kesari S (2011) Corticosteroids in brain cancer patients: benefits and pitfalls. Expert Rev Clin Pharmacol 4:233–242. 10.1586/ecp.11.1 21666852 10.1586/ecp.11.1 PMC3109638

[16] Dundar Y, Boland A, Strobl J, Dodd S, Haycox A, Bagust A, Bogg J, Dickson R, Walley T (2004) Newer hypnotic drugs for the short-term management of insomnia: a systematic review and economic evaluation. Health Technol Assess 8:iii–x, 1–125. 10.3310/hta8240 15193209 10.3310/hta8240

[17] Eibl T, Schrey M, Liebert A, Ritter L, Lange R, Steiner HH, Schebesch KM (2023) Influence of clinical and tumor-specific factors on the resting motor threshold in navigated transcranial magnetic stimulation. Neurophysiol Clin 53:102920. 10.1016/j.neucli.2023.102920 37944292 10.1016/j.neucli.2023.102920

[18] Engelhardt M, Komnenic D, Roth F, Kawelke L, Finke C, Picht T (2021) No impact of functional connectivity of the motor system on the resting motor threshold: a replication study. Front Neurosci 15:627445. 10.3389/fnins.2021.627445 33867916 10.3389/fnins.2021.627445 PMC8044353

[19] Engelhardt M, Schneider H, Gast T, Picht T (2019) Estimation of the resting motor threshold (RMT) in transcranial magnetic stimulation using relative-frequency and threshold-hunting methods in brain tumor patients. Acta Neurochir (Wien) 161:1845–1851. 10.1007/s00701-019-03997-z 31286238 10.1007/s00701-019-03997-z

[20] Fierro B, Salemi G, Brighina F, Buffa D, Conte S, La Bua V, Piazza A, Savettieri G (2002) A transcranial magnetic stimulation study evaluating methylprednisolone treatment in multiple sclerosis. Acta Neurol Scand 105:152–157. 10.1034/j.1600-0404.2002.1o369.x 11886356 10.1034/j.1600-0404.2002.1o369.x

[21] Fox J, Weisberg S (2019) An R companion to applied regression. Sage, Thousand Oaks

[22] Gerdelat-Mas A, Loubinoux I, Tombari D, Rascol O, Chollet F, Simonetta-Moreau M (2005) Chronic administration of selective serotonin reuptake inhibitor (SSRI) paroxetine modulates human motor cortex excitability in healthy subjects. Neuroimage 27:314–322. 10.1016/j.neuroimage.2005.05.009 16019236 10.1016/j.neuroimage.2005.05.009

[23] Gordon M, Lumley T (2017) forestplot: Advanced forest plot using ‘grid’graphics. R package version 1:70

[24] Groppa S, Oliviero A, Eisen A, Quartarone A, Cohen LG, Mall V, Kaelin-Lang A, Mima T, Rossi S, Thickbroom GW, Rossini PM, Ziemann U, Valls-Sole J, Siebner HR (2012) A practical guide to diagnostic transcranial magnetic stimulation: report of an IFCN committee. Clin Neurophysiol 123:858–882. 10.1016/j.clinph.2012.01.010 22349304 10.1016/j.clinph.2012.01.010 PMC4890546

[25] Hallett M (2007) Transcranial magnetic stimulation: a primer. Neuron 55:187–199. 10.1016/j.neuron.2007.06.026 17640522 10.1016/j.neuron.2007.06.026

[26] Harris PA, Taylor R, Minor BL, Elliott V, Fernandez M, O’Neal L, McLeod L, Delacqua G, Delacqua F, Kirby J, Duda SN, Consortium RE (2019) The REDCap consortium: building an international community of software platform partners. J Biomed Inform 95:103208. 10.1016/j.jbi.2019.103208 31078660 10.1016/j.jbi.2019.103208 PMC7254481

[27] Harris PA, Taylor R, Thielke R, Payne J, Gonzalez N, Conde JG (2009) Research electronic data capture (REDCap)–a metadata-driven methodology and workflow process for providing translational research informatics support. J Biomed Inform 42:377–381. 10.1016/j.jbi.2008.08.010 18929686 10.1016/j.jbi.2008.08.010 PMC2700030

[28] Heidegger T, Krakow K, Ziemann U (2010) Effects of antiepileptic drugs on associative LTP-like plasticity in human motor cortex. Eur J Neurosci 32:1215–1222. 10.1111/j.1460-9568.2010.07375.x 20726885 10.1111/j.1460-9568.2010.07375.x

[29] Herbsman T, Forster L, Molnar C, Dougherty R, Christie D, Koola J, Ramsey D, Morgan PS, Bohning DE, George MS, Nahas Z (2009) Motor threshold in transcranial magnetic stimulation: the impact of white matter fiber orientation and skull-to-cortex distance. Hum Brain Mapp 30:2044–2055. 10.1002/hbm.20649 18973261 10.1002/hbm.20649 PMC2893589

[30] Hotta N, Miyamoto M, Suzuki K (2022) Lamotrigine and retigabine increase motor threshold in transcranial magnetic stimulation at the dose required to produce an antiepileptic effect against maximal electroshock-induced seizure in rats. Neurosci Lett 771:136460. 10.1016/j.neulet.2022.136460 35051437 10.1016/j.neulet.2022.136460

[31] Ilic TV, Korchounov A, Ziemann U (2002) Complex modulation of human motor cortex excitability by the specific serotonin re-uptake inhibitor sertraline. Neurosci Lett 319:116–120. 10.1016/s0304-3940(01)02563-0 11825684 10.1016/s0304-3940(01)02563-0

[32] Ilic TV, Meintzschel F, Cleff U, Ruge D, Kessler KR, Ziemann U (2002) Short-interval paired-pulse inhibition and facilitation of human motor cortex: the dimension of stimulus intensity. J Physiol 545:153–167. 10.1113/jphysiol.2002.030122 12433957 10.1113/jphysiol.2002.030122 PMC2290644

[33] Inghilleri M, Berardelli A, Marchetti P, Manfredi M (1996) Effects of diazepam, baclofen and thiopental on the silent period evoked by transcranial magnetic stimulation in humans. Exp Brain Res 109:467–472. 10.1007/BF00229631 8817277 10.1007/BF00229631

[34] Inghilleri M, Conte A, Curra A, Frasca V, Lorenzano C, Berardelli A (2004) Ovarian hormones and cortical excitability. An rTMS study in humans. Clin Neurophysiol 115:1063–1068. 10.1016/j.clinph.2003.12.003 15066531 10.1016/j.clinph.2003.12.003

[35] Jaeger B (2016) r2glmm: Computes R squared for mixed (multilevel) models. CRAN: Contributed Packages

[36] Kappen PR, Kakar E, Dirven CMF, van der Jagt M, Klimek M, Osse RJ, Vincent A (2022) Delirium in neurosurgery: a systematic review and meta-analysis. Neurosurg Rev 45:329–341. 10.1007/s10143-021-01619-w 34396454 10.1007/s10143-021-01619-w PMC8827408

[37] Kimiskidis VK, Papagiannopoulos S, Kazis DA, Sotirakoglou K, Vasiliadis G, Zara F, Kazis A, Mills KR (2006) Lorazepam-induced effects on silent period and corticomotor excitability. Exp Brain Res 173:603–611. 10.1007/s00221-006-0402-1 16525803 10.1007/s00221-006-0402-1

[38] Kowski AB, Weissinger F, Gaus V, Fidzinski P, Losch F, Holtkamp M (2016) Specific adverse effects of antiepileptic drugs–a true-to-life monotherapy study. Epilepsy Behav 54:150–157. 10.1016/j.yebeh.2015.11.009 26709103 10.1016/j.yebeh.2015.11.009

[39] Kozel FA, Nahas Z, deBrux C, Molloy M, Lorberbaum JP, Bohning D, Risch SC, George MS (2000) How coil-cortex distance relates to age, motor threshold, and antidepressant response to repetitive transcranial magnetic stimulation. J Neuropsychiatry Clin Neurosci 12:376–384. 10.1176/jnp.12.3.376 10956572 10.1176/jnp.12.3.376

[40] Krieg SM, Buchmann NH, Gempt J, Shiban E, Meyer B, Ringel F (2012) Diffusion tensor imaging fiber tracking using navigated brain stimulation–a feasibility study. Acta Neurochir (Wien) 154:555–563. 10.1007/s00701-011-1255-3 22270529 10.1007/s00701-011-1255-3

[41] Krieg SM, Sollmann N, Obermueller T, Sabih J, Bulubas L, Negwer C, Moser T, Droese D, Boeckh-Behrens T, Ringel F, Meyer B (2015) Changing the clinical course of glioma patients by preoperative motor mapping with navigated transcranial magnetic brain stimulation. BMC Cancer 15:231. 10.1186/s12885-015-1258-1 25884404 10.1186/s12885-015-1258-1 PMC4423137

[42] Kuznetsova A, Brockhoff PB, Christensen RHB (2017) lmerTest Package: Tests in Linear Mixed Effects Models. Journal of Statistical Software 82. 10.18637/jss.v082.i13

[43] Lang N, Rothkegel H, Peckolt H, Deuschl G (2013) Effects of lacosamide and carbamazepine on human motor cortex excitability: a double-blind, placebo-controlled transcranial magnetic stimulation study. Seizure 22:726–730. 10.1016/j.seizure.2013.05.010 23778157 10.1016/j.seizure.2013.05.010

[44] Lauderdale K, Murphy T, Tung T, Davila D, Binder DK, Fiacco TA (2015) Osmotic edema rapidly increases neuronal excitability through activation of NMDA receptor-dependent slow inward currents in juvenile and adult hippocampus. ASN Neuro. 10.1177/1759091415605115 26489684 10.1177/1759091415605115 PMC4623564

[45] Lavrador JP, Gioti I, Hoppe S, Jung J, Patel S, Gullan R, Ashkan K, Bhangoo R, Vergani F (2020) Altered motor excitability in patients with diffuse gliomas involving motor eloquent areas: the impact of tumor grading. Neurosurgery 88:183–192. 10.1093/neuros/nyaa354 32888309 10.1093/neuros/nyaa354

[46] Loo CK, McFarquhar TF, Mitchell PB (2008) A review of the safety of repetitive transcranial magnetic stimulation as a clinical treatment for depression. Int J Neuropsychopharmacol 11:131–147. 10.1017/S1461145707007717 17880752 10.1017/S1461145707007717

[47] Louis DN, Ohgaki H, Wiestler OD, Cavenee WK, Burger PC, Jouvet A, Scheithauer BW, Kleihues P (2007) The 2007 WHO classification of tumours of the central nervous system. Acta Neuropathol 114:97–109. 10.1007/s00401-007-0243-4 17618441 10.1007/s00401-007-0243-4 PMC1929165

[48] Louis DN, Perry A, Reifenberger G, von Deimling A, Figarella-Branger D, Cavenee WK, Ohgaki H, Wiestler OD, Kleihues P, Ellison DW (2016) The 2016 World Health Organization classification of tumors of the central nervous system: a summary. Acta Neuropathol 131:803–820. 10.1007/s00401-016-1545-1 27157931 10.1007/s00401-016-1545-1

[49] Lynch BA, Lambeng N, Nocka K, Kensel-Hammes P, Bajjalieh SM, Matagne A, Fuks B (2004) The synaptic vesicle protein SV2A is the binding site for the antiepileptic drug levetiracetam. Proc Natl Acad Sci U S A 101:9861–9866. 10.1073/pnas.0308208101 15210974 10.1073/pnas.0308208101 PMC470764

[50] Mattheisen GB, Tsintsadze T, Smith SM (2018) Strong G-protein-mediated inhibition of sodium channels. Cell Rep 23:2770–2781. 10.1016/j.celrep.2018.04.109 29847805 10.1016/j.celrep.2018.04.109 PMC6203318

[51] Mbizvo GK, Dixon P, Hutton JL, Marson AG (2013) The adverse effects profile of levetiracetam in epilepsy: a more detailed look. Int J Neurosci 124:627–634. 10.3109/00207454.2013.866951 24256446 10.3109/00207454.2013.866951

[52] McConnell KA, Nahas Z, Shastri A, Lorberbaum JP, Kozel FA, Bohning DE, George MS (2001) The transcranial magnetic stimulation motor threshold depends on the distance from coil to underlying cortex: a replication in healthy adults comparing two methods of assessing the distance to cortex. Biol Psychiatry 49:454–459. 10.1016/s0006-3223(00)01039-8 11274657 10.1016/s0006-3223(00)01039-8

[53] McDonnell MN, Zipser C, Darmani G, Ziemann U, Muller-Dahlhaus F (2018) The effects of a single dose of fluoxetine on practice-dependent plasticity. Clin Neurophysiol 129:1349–1356. 10.1016/j.clinph.2018.04.604 29729588 10.1016/j.clinph.2018.04.604

[54] Menzler K, Hermsen A, Balkenhol K, Duddek C, Bugiel H, Bauer S, Schorge S, Reif PS, Klein KM, Haag A, Oertel WH, Hamer HM, Knake S, Trucks H, Sander T, Rosenow F, Consortium E (2014) A common SCN1A splice-site polymorphism modifies the effect of carbamazepine on cortical excitability–a pharmacogenetic transcranial magnetic stimulation study. Epilepsia 55:362–369. 10.1111/epi.12515 24417206 10.1111/epi.12515

[55] Milani P, Piu P, Popa T, della Volpe R, Bonifazi M, Rossi A, Mazzocchio R (2010) Cortisol-induced effects on human cortical excitability. Brain Stimul 3:131–139. 10.1016/j.brs.2009.07.004 20633442 10.1016/j.brs.2009.07.004

[56] Minelli A, Bortolomasi M, Scassellati C, Salvoro B, Avesani M, Manganotti P (2010) Effects of intravenous antidepressant drugs on the excitability of human motor cortex: a study with paired magnetic stimulation on depressed patients. Brain Stimul 3:15–21. 10.1016/j.brs.2009.04.003 20633426 10.1016/j.brs.2009.04.003

[57] Moritz I, Engelhardt M, Rosenstock T, Grittner U, Schweizerhof O, Khakhar R, Schneider H, Mirbagheri A, Zdunczyk A, Faust K, Vajkoczy P, Picht T (2024) Preoperative nTMS analysis: a sensitive tool to detect imminent motor deficits in brain tumor patients. Acta Neurochir (Wien) 166:419. 10.1007/s00701-024-06308-3 39432031 10.1007/s00701-024-06308-3 PMC11493810

[58] Moser I, Engelhardt M, Grittner U, Ferreira F, Denker M, Reinsch J, Fischer L, Link T, Heppner FL, Capper D, Vajkoczy P, Picht T, Rosenstock T (2025) Analysis of neuronal excitability profiles for motor-eloquent brain tumor entities using nTMS in 800 patients. Cancers (Basel). 10.3390/cancers17060935 40149270 10.3390/cancers17060935 PMC11940777

[59] Muller V, Birbaumer N, Preissl H, Braun C, Lang F (2002) Effects of water on cortical excitability in humans. Eur J Neurosci 15:528–538. 10.1046/j.0953-816x.2001.01886.x 11876780 10.1046/j.0953-816x.2001.01886.x

[60] Oldfield RC (1971) The assessment and analysis of handedness: the Edinburgh inventory. Neuropsychologia 9:97–113. 10.1016/0028-3932(71)90067-4 5146491 10.1016/0028-3932(71)90067-4

[61] Oliviero A, Profice P, Tonali PA, Pilato F, Saturno E, Dileone M, Ranieri F, Di Lazzaro V (2006) Effects of aging on motor cortex excitability. Neurosci Res 55:74–77. 10.1016/j.neures.2006.02.002 16584795 10.1016/j.neures.2006.02.002

[62] Palmieri MG, Iani C, Scalise A, Desiato MT, Loberti M, Telera S, Caramia MD (1999) The effect of benzodiazepines and flumazenil on motor cortical excitability in the human brain. Brain Res 815:192–199. 10.1016/s0006-8993(98)01164-0 9878733 10.1016/s0006-8993(98)01164-0

[63] Paulus W, Classen J, Cohen LG, Large CH, Di Lazzaro V, Nitsche M, Pascual-Leone A, Rosenow F, Rothwell JC, Ziemann U (2008) State of the art: pharmacologic effects on cortical excitability measures tested by transcranial magnetic stimulation. Brain Stimul 1(3):151–163. 10.1016/j.brs.2008.06.002 20633382 10.1016/j.brs.2008.06.002

[64] Peinemann A, Lehner C, Conrad B, Siebner HR (2001) Age-related decrease in paired-pulse intracortical inhibition in the human primary motor cortex. Neurosci Lett 313:33–36. 10.1016/s0304-3940(01)02239-x 11684333 10.1016/s0304-3940(01)02239-x

[65] Pitcher JB, Ogston KM, Miles TS (2003) Age and sex differences in human motor cortex input-output characteristics. J Physiol 546:605–613. 10.1113/jphysiol.2002.029454 12527746 10.1113/jphysiol.2002.029454 PMC2342521

[66] Powell G, Logan J, Kiri V, Borghs S (2019) Trends in antiepileptic drug treatment and effectiveness in clinical practice in England from 2003 to 2016: a retrospective cohort study using electronic medical records. BMJ Open 9:e032551. 10.1136/bmjopen-2019-032551 31848168 10.1136/bmjopen-2019-032551 PMC6936987

[67] Premoli I, Biondi A, Carlesso S, Rivolta D, Richardson MP (2017) Lamotrigine and levetiracetam exert a similar modulation of TMS-evoked EEG potentials. Epilepsia 58:42–50. 10.1111/epi.13599 27808418 10.1111/epi.13599 PMC5244669

[68] Premoli I, Costantini A, Rivolta D, Biondi A, Richardson MP (2017) The effect of Lamotrigine and Levetiracetam on TMS-evoked EEG responses depends on stimulation intensity. Front Neurosci 11:585. 10.3389/fnins.2017.00585 29104528 10.3389/fnins.2017.00585 PMC5655014

[69] Pridmore S, Morey R, Rybak M (2021) Variability in motor threshold. Brain Stimul 14:1259–1260. 10.1016/j.brs.2021.08.013 34416370 10.1016/j.brs.2021.08.013

[70] R Core Team (2024) R: A Language and Environment for Statistical Computing. R Foundation for Statistical Computing, Vienna, Austria. https://www.R-project.org/.

[71] Raffa G, Scibilia A, Conti A, Ricciardo G, Rizzo V, Morelli A, Angileri FF, Cardali SM, Germano A (2019) The role of navigated transcranial magnetic stimulation for surgery of motor-eloquent brain tumors: a systematic review and meta-analysis. Clin Neurol Neurosurg 180:7–17. 10.1016/j.clineuro.2019.03.003 30870762 10.1016/j.clineuro.2019.03.003

[72] Reis J, Wentrup A, Hamer HM, Mueller HH, Knake S, Tergau F, Oertel WH, Rosenow F (2004) Levetiracetam influences human motor cortex excitability mainly by modulation of ion channel function–a TMS study. Epilepsy Res 62:41–51. 10.1016/j.eplepsyres.2004.08.001 15519131 10.1016/j.eplepsyres.2004.08.001

[73] Robol E, Fiaschi A, Manganotti P (2004) Effects of citalopram on the excitability of the human motor cortex: a paired magnetic stimulation study. J Neurol Sci 221:41–46. 10.1016/j.jns.2004.03.007 15178212 10.1016/j.jns.2004.03.007

[74] Rosenstock T, Grittner U, Acker G, Schwarzer V, Kulchytska N, Vajkoczy P, Picht T (2017) Risk stratification in motor area-related glioma surgery based on navigated transcranial magnetic stimulation data. J Neurosurg 126:1227–1237. 10.3171/2016.4.JNS152896 27257834 10.3171/2016.4.JNS152896

[75] Rosenstock T, Hani L, Grittner U, Schlinkmann N, Ivren M, Schneider H, Raabe A, Vajkoczy P, Seidel K, Picht T (2022) Bicentric validation of the navigated transcranial magnetic stimulation motor risk stratification model. J Neurosurg 136:1194–1206. 10.3171/2021.3.JNS2138 34534966 10.3171/2021.3.JNS2138

[76] Rossini PM, Barker AT, Berardelli A, Caramia MD, Caruso G, Cracco RQ, Dimitrijević MR, Hallett M, Katayama Y, Lücking CH, Maertens de Noordhout AL, Marsden CD, Murray NMF, Rothwell JC, Swash M, Tomberg C (1994) Non-invasive electrical and magnetic stimulation of the brain, spinal cord and roots: basic principles and procedures for routine clinical application. Report of an IFCN committee. Electroencephalogr Clin Neurophysiol 91:79–92. 10.1016/0013-4694(94)90029-9 7519144 10.1016/0013-4694(94)90029-9

[77] Rossini PM, Burke D, Chen R, Cohen LG, Daskalakis Z, Di Iorio R, Di Lazzaro V, Ferreri F, Fitzgerald PB, George MS, Hallett M, Lefaucheur JP, Langguth B, Matsumoto H, Miniussi C, Nitsche MA, Pascual-Leone A, Paulus W, Rossi S, Rothwell JC, Siebner HR, Ugawa Y, Walsh V, Ziemann U (2015) Non-invasive electrical and magnetic stimulation of the brain, spinal cord, roots and peripheral nerves: basic principles and procedures for routine clinical and research application. An updated report from an I.F.C.N. committee. Clin Neurophysiol 126:1071–1107. 10.1016/j.clinph.2015.02.001 25797650 10.1016/j.clinph.2015.02.001 PMC6350257

[78] Rozand V, Senefeld JW, Sundberg CW, Smith AE, Hunter SK (2019) Differential effects of aging and physical activity on corticospinal excitability of upper and lower limb muscles. J Neurophysiol 122:241–250. 10.1152/jn.00077.2019 31091158 10.1152/jn.00077.2019 PMC6689774

[79] Schonle PW, Isenberg C, Crozier TA, Dressler D, Machetanz J, Conrad B (1989) Changes of transcranially evoked motor responses in man by midazolam, a short acting benzodiazepine. Neurosci Lett 101:321–324. 10.1016/0304-3940(89)90553-3 2771175 10.1016/0304-3940(89)90553-3

[80] Shibuya K, Park SB, Geevasinga N, Huynh W, Simon NG, Menon P, Howells J, Vucic S, Kiernan MC (2016) Threshold tracking transcranial magnetic stimulation: effects of age and gender on motor cortical function. Clin Neurophysiol 127:2355–2361. 10.1016/j.clinph.2016.03.009 27178853 10.1016/j.clinph.2016.03.009

[81] ShinyMeta (2021) forestplotter: Create Forest Plots in Shiny and R. GitHub repository. https://github.com/ShinyMeta/forestplotter

[82] Siebner HR, Ziemann U (2007) Das TMS-Buch. Springer, Berlin, Heidelberg. 10.1007/978-3-540-71905-2

[83] Silbert BI, Pevcic DD, Patterson HI, Windnagel KA, Thickbroom GW (2013) Inverse correlation between resting motor threshold and corticomotor excitability after static magnetic stimulation of human motor cortex. Brain Stimul 6:817–820. 10.1016/j.brs.2013.03.007 23598254 10.1016/j.brs.2013.03.007

[84] Silbert LC, Nelson C, Holman S, Eaton R, Oken BS, Lou JS, Kaye JA (2006) Cortical excitability and age-related volumetric MRI changes. Clin Neurophysiol 117:1029–1036. 10.1016/j.clinph.2006.02.003 16564739 10.1016/j.clinph.2006.02.003

[85] Smith MJ, Adams LF, Schmidt PJ, Rubinow DR, Wassermann EM (2002) Effects of ovarian hormones on human cortical excitability. Ann Neurol 51:599–603. 10.1002/ana.10180 12112106 10.1002/ana.10180

[86] Smith MJ, Keel JC, Greenberg BD, Adams LF, Schmidt PJ, Rubinow DA, Wassermann EM (1999) Menstrual cycle effects on cortical excitability. Neurology 53:2069–2072. 10.1212/wnl.53.9.2069 10599783 10.1212/wnl.53.9.2069

[87] Sohn YH, Kaelin-Lang A, Jung HY, Hallett M (2001) Effect of levetiracetam on human corticospinal excitability. Neurology 57:858–863. 10.1212/wnl.57.5.858 11552017 10.1212/wnl.57.5.858

[88] Solinas C, Lee YC, Reutens DC (2008) Effect of levetiracetam on cortical excitability: a transcranial magnetic stimulation study. Eur J Neurol 15:501–505. 10.1111/j.1468-1331.2008.02110.x 18394048 10.1111/j.1468-1331.2008.02110.x

[89] Sollmann N, Tanigawa N, Bulubas L, Sabih J, Zimmer C, Ringel F, Meyer B, Krieg SM (2017) Clinical Factors Underlying the Inter-individual Variability of the Resting Motor Threshold in Navigated Transcranial Magnetic Stimulation Motor Mapping. Brain Topogr 30:98–121. 10.1007/s10548-016-0536-9 27815647 10.1007/s10548-016-0536-9

[90] Sollmann N, Zhang H, Fratini A, Wildschuetz N, Ille S, Schroder A, Zimmer C, Meyer B, Krieg SM (2020) Risk assessment by presurgical tractography using navigated TMS maps in patients with highly motor- or language-eloquent brain tumors. Cancers (Basel). 10.3390/cancers12051264 32429502 10.3390/cancers12051264 PMC7281396

[91] Sommer M, Gileles E, Knappmeyer K, Rothkegel H, Polania R, Paulus W (2012) Carbamazepine reduces short-interval interhemispheric inhibition in healthy humans. Clin Neurophysiol 123:351–357. 10.1016/j.clinph.2011.07.027 21862399 10.1016/j.clinph.2011.07.027

[92] Soong C, Ethier C, Lee Y, Othman D, Burry L, Wu PE, Ng KA, Matelski J, Liu B (2022) Reducing sedative-hypnotics among hospitalized patients: a multi-centered study. J Gen Intern Med 37:2345–2350. 10.1007/s11606-021-07292-5 34981347 10.1007/s11606-021-07292-5 PMC9360352

[93] Stokes MG, Chambers CD, Gould IC, Henderson TR, Janko NE, Allen NB, Mattingley JB (2005) Simple metric for scaling motor threshold based on scalp-cortex distance: application to studies using transcranial magnetic stimulation. J Neurophysiol 94:4520–4527. 10.1152/jn.00067.2005 16135552 10.1152/jn.00067.2005

[94] Tergau F, Wischer S, Somal HS, Nitsche MA, Mercer AJ, Paulus W, Steinhoff BJ (2003) Relationship between lamotrigine oral dose, serum level and its inhibitory effect on CNS: insights from transcranial magnetic stimulation. Epilepsy Res 56:67–77. 10.1016/j.eplepsyres.2003.08.006 14529954 10.1016/j.eplepsyres.2003.08.006

[95] van der Meer PB, Dirven L, van den Bent MJ, Preusser M, Taphoorn MJB, Ruda R, Koekkoek JAF (2022) Prescription preferences of antiepileptic drugs in brain tumor patients: An international survey among EANO members. Neurooncol Pract 9:105–113. 10.1093/nop/npab059 35371521 10.1093/nop/npab059 PMC8965049

[96] Veldema J, Bosl K, Nowak DA (2017) Motor recovery of the affected hand in subacute stroke correlates with changes of contralesional cortical hand motor representation. Neural Plast 2017:6171903. 10.1155/2017/6171903 28286677 10.1155/2017/6171903 PMC5329670

[97] Veldema J, Nowak DA, Gharabaghi A (2021) Resting motor threshold in the course of hand motor recovery after stroke: a systematic review. J Neuroeng Rehabil 18:158. 10.1186/s12984-021-00947-8 34732203 10.1186/s12984-021-00947-8 PMC8564987

[98] Wang W, Lin Y (2023) Fundamentals of Magnetic Stimulation Devices. Therapeutics of Neural Stimulation for Neurological Disorders. Springer, Singapore, pp 133–154. 10.1007/978-981-99-4538-2_8

[99] Wassermann EM (1998) Risk and safety of repetitive transcranial magnetic stimulation: report and suggested guidelines from the International Workshop on the Safety of Repetitive Transcranial Magnetic Stimulation, June 5–7, 1996. Electroencephalography and Clinical Neurophysiology/Evoked Potentials Section 108:1-16. 10.1016/s0168-5597(97)00096-8 10.1016/s0168-5597(97)00096-8 9474057

[100] Wassermann EM (2002) Variation in the response to transcranial magnetic brain stimulation in the general population. Clin Neurophysiol 113:1165–1171. 10.1016/s1388-2457(02)00144-x 12088713 10.1016/s1388-2457(02)00144-x

[101] Werhahn KJ, Forderreuther S, Straube A (1998) Effects of the serotonin1B/1D receptor agonist zolmitriptan on motor cortical excitability in humans. Neurology 51:896–898. 10.1212/wnl.51.3.896 9748054 10.1212/wnl.51.3.896

[102] Wickham H, Averick M, Bryan J, Chang W, McGowan L, François R, Grolemund G, Hayes A, Henry L, Hester J, Kuhn M, Pedersen T, Miller E, Bache S, Müller K, Ooms J, Robinson D, Seidel D, Spinu V, Takahashi K, Vaughan D, Wilke C, Woo K, Yutani H (2019) Welcome to the tidyverse. J Open Source Softw. 10.21105/joss.01686

[103] Young-Bernier M, Davidson PS, Tremblay F (2012) Paired-pulse afferent modulation of TMS responses reveals a selective decrease in short latency afferent inhibition with age. Neurobiol Aging 33(835):e831–e811. 10.1016/j.neurobiolaging.2011.08.012 10.1016/j.neurobiolaging.2011.08.012 21958964

[104] Ziemann U, Lonnecker S, Steinhoff BJ, Paulus W (1996) The effect of lorazepam on the motor cortical excitability in man. Exp Brain Res 109:127–135. 10.1007/BF00228633 8740215 10.1007/BF00228633

[105] Ziemann U, Lonnecker S, Steinhoff BJ, Paulus W (1996) Effects of antiepileptic drugs on motor cortex excitability in humans: a transcranial magnetic stimulation study. Ann Neurol 40:367–378. 10.1002/ana.410400306 8797526 10.1002/ana.410400306

[106] Ziemann U, Reis J, Schwenkreis P, Rosanova M, Strafella A, Badawy R, Muller-Dahlhaus F (2015) TMS and drugs revisited 2014. Clin Neurophysiol 126:1847–1868. 10.1016/j.clinph.2014.08.028 25534482 10.1016/j.clinph.2014.08.028

[107] Zona C, Niespodziany I, Marchetti C, Klitgaard H, Bernardi G, Margineanu DG (2001) Levetiracetam does not modulate neuronal voltage-gated Na+ and T-type Ca2+ currents. Seizure 10:279–286. 10.1053/seiz.2000.0504 11466024 10.1053/seiz.2000.0504

